# Medical and Philosophical Intelligence

**Published:** 1815-04

**Authors:** 


### MEDICAL AND PHILOSOPHICAL INTELLIGENCE.

ON the 8th of March was the Anniversary Meeting of the *Medical Society of London*. The oration, delivered by Mr. TAUNTON, comprehended a succinct but complete view of the various kinds of hernia, and the methods of treatment, whether by operation or by artificially retaining the contents of the abdomen or pelvis. Specimens of various trusses were exhibited, and the advantages and disadvantages of each pointed out.

The following were chosen officers for the ensuing year:—President, Dr. Joseph Adams; Vice-Presidents, Dr. Clutterbuck, Dr. Babington, Mr. Abernethy, and Mr. Norris; Treasurer, Mr. Andree; Librarian, Dr. Hancock; Secretaries, Dr. Fothergill and Mr. Blegborough; ditto foreign correspondence, Dr. Taylor; and Registrar, Mr. Pettigrew; Orator for the ensuing year, Dr. Clutterbuck.

---

*Apothecaries' Hall*, *Feb.* 20, 1815.—The Society of Apothecaries having completed several new arrangements in their laboratories, in which steam is employed for the purposes of evaporation, distillation, &c. such professional gentlemen as are desirous of viewing these improvements are informed, that they will be open for their inspection on the second Tuesday in March, April, May, and June, between the hours of two and three o'clock in the afternoon.

There

The Society of Apothecaries have published an outline of their Bill before Parliament, and a Statement of the Motives which have induced them to apply for such Bill. These we shall give in our next.

---

The following being a circular letter sent to the county members of Berks, and also to the members of each borough-town in the county, we are requested to insert it in our Journal.

*Crown-Inn, Reading*; *March* 22, 1815.

SIR,—A meeting of the medical gentlemen of the county being held here by public advertisement, for the purpose of taking into consideration the propriety of petitioning Parliament on the subject of the proposed additional Assessment on Horses, Carriages, and Servants; it was communicated to them, that similar Petitions had not been received, being contrary to a standing order in the Houses pending a Money Bill.

The meeting respectfully solicit your particular attention to this subject, requesting, that, when it comes under the consideration of Parliament, you would have the goodness to endeavour to obtain for them that relief which their peculiar situation requires.

(Signed, by the order of the meeting,) WIDDOWS GOLDING, Chairman.

P.S. It may not be amiss to state, that, in the late Tax of "Treble Assessment," Mr. Pitt thought it right to afford Medical Practitioners relief on Horses and Carriages.

---

M. DONOVAN, secretary to the Kirwanian Society in Dublin, has read a paper, in which he endeavours to shew that the principles of Galvanism and Electricity are different, the former being more immediately connected with chemical affinity than the latter. We shall, for the future, avoid as much as possible anticipating the crude accounts collected from the different societies, reserving our opinions till their transactions are published. On the present occasion, however, we believe most of our readers will join in the opinion that there has always appeared something in the laws of these two phenomena not strictly reconcileable to each other, though in many respects they accord so perfectly.

---

Dr. AYRTON PARIS, who has settled at Penzance, to whom we are obliged for a valuable practical treatise on Pharmaceutic Chemistry, has availed himself of his chemical knowledge in his new situation, and established a Geological Society for the county of Cornwall, abounding in minerals, some of which are with difficulty met with in any other part of the world. The Prince Regent has become its patron.

"It was resolved, that a deputation should present an address of thanks to his Royal Highness, for the great honour conferred upon them; and that it should consist of the Vice Patrons, Lord De Dunstanville, and the Earl of Yarmouth; the President, Davies Giddy, Esq. M.P.; and its founder, Dr. Ayrton Paris.

"Apartments have been provided at Penzance, which contain a collection of minerals already highly interesting: among the later additions we may notice *rutilute*, lately discovered in the slate quarries at Tintagel; *a grey copper ore* from Crennis mine, the composition of which resembles the *fal-erz*, with the exception of lead, (on the authority of the Rev. William Gregor.) *Wood tin* from from Trethurgy Moor, near St. Austel, in a matrix of shorl and quartz. *The triple sulphuret of antimony, lead, and copper*, which has re-appeared at the antimony mine, near Port Isaac, after a lapse of twenty years.—*Sulphate of barytes,* now found at Huel Unity, for the first time in Cornwall.—A large quantity of *stream gold,* presented by Sir Christopher Hawkins, with an interesting account of its discovery, in which he states that it was found in streaming for tin in a moor in the parish of Ladock; and offers some information, which he trusts may direct future adventurers to a successful undertaking.—Many other communications have been also read before the society, which we shall notice as soon as they appear, as we understand the society are preparing a volume of Transactions, which are shortly to be published."

---

Another instance has occurred of Hydrophobia in St. Bartholomew's Hospital. The patient was brought to the house immediately after the accident, but either the dog was not suspected to be rabid, or the part bitten was so situated as to render amputation impracticable. At the end of about six weeks the symptoms appeared. Venesection was tried to a considerable extent, but without any advantage.

---

There has been and still exists an epidemic fever in Cambridgeshire, which has penetrated the University, where it has proved fatal to some of the students. We are promised a communication on the subject, and shall be thankful for others.

---

Dr. William Cullen Brown, of 50, Rathbone-Place, son of the distinguished author of the Brunonian system, has announced his intention of giving instructions to medical students and practitioners who may require assistance in the literary part of their education.

---

We have been informed, that a new edition of the Pharmacopœia Londinensis is in great forwardness. The college, it seems, has condescended to consult some practical chemists on the occasion; we may therefore hope for a less imperfect production than the one now in use, the inaccuracies of which have been so severely animadverted upon in various publications. At the request of the college committee, Mr. Hume, of Long Acre, has examined several of the formulæ, particularly that for Antimon. Tartaris. for which that ingenious chemist has devised some new processes, one of which, we understand, is approved of and will appear in the new edition. It is prepared from the black sulphuret of antimony, which is decomposed by nitrate of potash and sulphuric acid. The process is attended with no difficulty and little expence; and the emetic tartar produced is perfect in the figure of its crystals, and in all respects extremely neat and pure, being as white as the super-tartrate of potash itself, and in efficacy not to be surpassed.

---

*Chameleon, Colours, Bats, Parrots, Monkeys.*—Mr. Forbes (in his Oriental Memoirs, an interesting and splendid work), when at Dažagan in Concan, then belonging to the Mahrattas, kept a chameleon for several weeks, and paid great attention to its changing colours. Its general colour was "a pleasant green," spotted with pale blue. Its customary changes were to a bright yellow, a dark olive, and a dull green; but, when irritated, or when a dog approached, in which case fear was perhaps the operating cause, the body became considerably inflated, and the skin clouded like tortoiseshell, in shades of yellow, orange, green, and black: in these circumstances it appeared to most advantage. The animal was most singularly affected by any thing black: the skirting-board of the room was black, and the creature carefully avoided it; but if, by chance, he came near it, or if a black hat were placed in his way, he shrunk to a skeleton and became black as jet. It was evident, by the care he took to avoid those objects which occasioned this change, that it was painful to him. The colour seemed to operate like a poison.

Obliged one night to take up his quarters in the tomb-chambers of a Mahomedan grave (for the houses were not to be defiled by admitting a Christian), Mr. Forbes had first to drive out the previous occupiers—some enormous bats, from their size denominated "flying foxes." Those animals frequently measure six feet from wing to wing.

On the coast where Angengo is situated, the parrots are as much dreaded at the time of harvest, as a flight of locusts, or a desolating Mahratta army. They darken the air by their numbers, and when they alight in a rice-field carry off every grain in a few hours.

At Dhuboy, the capital of a district containing 84 villages, of which Mr. Forbes was appointed governor (after its surrender to General Goddard in 1780), he found as many monkeys as human beings. They seemed to have full possession of all the roofs of the houses; and they are sometimes called in as auxiliaries in disputes between neighbours. In quarrels of this kind they never have recourse to blows, but employ every kind of invective against each other and against all their relatives. The person who is worsted in this war of words, frequently takes an opportunity to strew some rice over the roof of his adversary's house. The monkeys soon discover this treasure, and resort in crowds to pick it up: when finding that much of it has fallen between the tiles (which are not nailed down as with us), they make no scruple to remove them, and nearly unroof the house. When workmen cannot be procured to repair the roof, rain is admitted, which ruins the furniture and the stock of grain.

---

*Announced Metallization of Charcoal.*—Mr. Dobereiner, Professor of Chemistry of the University of Jena, in Saxony, informs Mr. Accum, that he has discovered charcoal to be a *metallic compound.* The following lines he has received from Mr. Dobereiner on this subject:—

............... "I hope to be able to communicate to you the successful metallization of charcoal, which I have found contains tains a metallic base. In cast iron and in steel the metal is present in a metallic state, and may be separated from both of them by the united action of phosphorus and an alkali. Further particulars, concerning this subject, I will transmit to you in my next.

"*To Mr. Accum, London.* DOBEREINER."

---

The following statement respecting the City or Royal Hospitals was read at the Mansion House:—

CHRIST'S HOSPITAL.

| | |
|---|---|
| Children put forth Apprentices | 162 |
| Children buried last year | 6 |
| Children now under the care of the Hospital at Hertford and London | 1,053 |
| To be admitted on Presentation | 130 |

ST. BARTHOLOMEW'S HOSPITAL.

| | |
|---|---|
| Patients admitted, cured, and discharged last year | 9,236 |
| Buried last year | 288 |
| Remained under cure—In-patients | 407 |
| ——————Out-patients | 215 |
| So there has been under the charge of the Hospital last year | 10,146 |

ST. THOMAS'S HOSPITAL.

| | |
|---|---|
| Cured and discharged last year—In-patients | 2769 |
| ——————Out-patients | 6323 |
| Remaining under cure—In-patients | 411 |
| ——————Out-patients | 316 |
| So there has been under the charge of the Hospital last year | 9,994 |

BRIDEWELL HOSPITAL.

| | |
|---|---|
| Vagrants committed by the Lord Mayor and sitting Aldermen | 240 |
| Paupers to be sent to their Parishes | 558 |
| Apprentices now in the Hospital, brought up to divers trades | 35 |

BETHLEM HOSPITAL.

| | | |
|---|---|---|
| Distracted Men and Women remaining Dec. 31, 1813 | | 143 |
| Admitted during the year 1814 | | 93 |
| Cured and discharged | 109 | |
| Buried | 8 | |
| Remaining under cure | 119 | |

---

Dr. SPURZHEIM recommenced his Lectures on Friday last.

Dr. DAVIS will commence his Summer Course of Lectures on the Theory and Practice of Midwifery, on Monday, the 24th of April, at his house, 18, Lower Charlotte-street, Bedford-square.

Dr. MERRIMAN's Course of Lectures on Midwifery, &c. will re-commence at the Middlesex Hospital, on Monday, April 10th.

---

*To the Editor of the Medical and Physical Journal.*

SIR,

HAVING inserted a letter in the last Number of your Journal from Mr. Walker, in justice, as you say, to that gentleman, I have to request a similar favour in justice to myself.

The reason for my omitting the words "because it is the most familiar familiar instance of fermentation," or in the language of Mr. Walker, "*mutilating*" his illustration, was, that I conceived they had nothing to do with the point at issue. All that was requisite was, that the example adduced should be appropriate; and, if it were not, its being familiar would not render it so. Does the supplying of the passage omitted make any thing more for the existence of Mr. Walker's *essence?* I must allow with you, Sir, that protracted controversies even on practical subjects become irksome; how much more must those become which are purely hypothetical? Mr. Walker clings, however, to the last, to his beloved essence, as a fond parent to the corse of his departed offspring. *Requiescat in pace.* I am, &c. JAS. WOODHAM.

*March* 2, 1815.

### REPORT OF DISEASES.

THE Chronic Cough and Dyspnœa, generally so prevalent at this season of the year, has much declined. Some cases of Acute Pulmonic Inflammation, however, have occurred. In two instances, the pain was inconsiderable, and the symptoms did not appear to the patients or their friends to indicate any danger, until very near the termination of the disease, when the physician who was called in had no hesitation in pronouncing the complaint to be too far advanced for art to relieve. One of the patients was an elderly woman, the other an infant; and both were probably lost for want of bleeding in the commencement of the disease.

Pulmonary Consumption, in spite of the mildness of the weather, has been frequent. In this disease, cold does not seem to be so prejudicial as the atmospheric variations, which occur in our climate even in a mild season. But there are other causes combining in the production of this disease, too much neglected by physicians, and often entirely lost sight of in the treatment. The subjects of consumption are generally of a tender delicate habit, easily affected by external agents, and often endowed with a sort of morbid sensibility. Their minds are excited by the exhilarating passions, or depressed by slight disappointments; and the circulation and respiration are hurried by the most trifling occurrences. In such cases, a high temperature will rather tend to accelerate than to retard the progress of the disease. The medicines which in a few solitary instances have proved efficacious, are emetics and fox-glove, and these seem to produce the beneficial effect by acting on the sanguineous system and reducing the velocity of the pulse. It is, therefore, a legitimate inference, that whatever produces a contrary effect is prejudicial. If the febrile state and frequency of the pulse be increased, whether by external heat or mental emotion, the effect is bad, and the exciting cause should, if possible, be removed. But heat has a direct and immediate effect on the pulse, and the long-continued application of it renders the mind irritable and the body more susceptible of disease. The variation of the seasons is conducive to health, both by relieving the mind from lassitude, or rather ennui, into which it is apt to degenerate from a constant sameness of weather; and the body is invigorated and refreshed by the salutary changes of atmosphere which nature has prescribed. These, however, have their limitations; and in disease it becomes the physician to observe how he can modify and direct them to advantage. If some patients have received benefit from being confined during the winter months in heated apartments, others have regained their health under very opposite circumstances. As far as the Reporter's own experience extends, females seem to bear confinement better than males, which may readily be accounted for from their constitutional difference.

LONDON

### LONDON PRICES OF DRUGS.—March 23, 1815.

| | £ | s | d. | £. | s. | d. per |
|---|---|---|---|---|---|---|
| Aloes Barbadoes, from | 22 | 0 | 0 to | 0 | 0 | 0 C. |
| ——Cape | 6 | 10 | 0.. | 7 | 0 | 0 — |
| ——Succotrina | 23 | 0 | 0.. | 0 | 0 | 0 — |
| ——Epatica or E.I. | 14 | 0 | 0.. | 16 | 0 | 0 — |
| Angelica Root | 7 | 10 | 0.. | 8 | 0 | 0 — |
| Alkanet Root | 5 | 10 | 0.. | 6 | 0 | 0 — |
| Antimony Crude | 2 | 18 | 0.. | 3 | 5 | 0 — |
| Aqua Fortis | S. | 0 | 8.. | D. | 1 | 1 lb. |
| Arrow Root, fine | 0 | 2 | 0.. | 0 | 3 | 6 — |
| ——ordinary | 0 | 0 | 9.. | 0 | 1 | 3 — |
| Arsenic, Red | 4 | 4 | 0.. | 4 | 10 | 0 C. |
| —— White | 2 | 16 | 0.. | 0 | 0 | 0 — |
| Balsam Capivi | 0 | 4 | 10.. | 0 | 5 | 0 lb. |
| —— Peru | 1 | 0 | 0.. | 1 | 1 | 0 — |
| —— Tolu | 0 | 14 | 6.. | 0 | 15 | 0 — |
| Bark Jesuits', Com. | 0 | 3 | 6.. | 0 | 4 | 0 — |
| ——Second | 0 | 6 | 0.. | 0 | 7 | 0 — |
| ——Quil or best | 0 | 8 | 0.. | 0 | 10 | 0 — |
| ——Red | 0 | 8 | 8.. | 0 | 9 | 0 — |
| ——Yellow | 0 | 2 | 0.. | 0 | 2 | 2 — |
| Borax, refined E.I. | none. | | | | | |
| ——English | 0 | 2 | 8.. | 0 | 3 | 0 lb. |
| ——unrefined or Tinc. | 6 | 10 | 0.. | 7 | 15 | 0 C. |
| Camphire, refined | 0 | 6 | 0.. | 0 | 6 | 3 lb. |
| —— unrefined | 16 | 0 | 0.. | 17 | 10 | 0 C. |
| Cantharides | 0 | 10 | 6.. | 0 | 11 | 6 lb. |
| Cardamoms (best) | 0 | 6 | 6.. | 0 | 7 | 6 — |
| Cassia Buds | 32 | 0 | 0 | 0 | 0 | 0 C. |
| —— Fistula, W.I. | 5 | 5 | 0 | in bond | | — |
| —— Lignea | 42 | 0 | 0 | 45 | 0 | 0 — |
| Castoreum American | 2 | 10 | 0.. | 3 | 0 | 0 lb. |
| —— Russia | 11 | 11 | 0.. | 0 | 0 | 0 — |
| Castor Oil, per bottle 1½ lb. | 0 | 3 | 9.. | 0 | 4 | 0 bo |
| Cocculus Indicus | 6 | 0 | 0.. | 7 | 0 | 0 C. |
| Colocynth Turkey | 0 | 5 | 0.. | 0 | 5 | 9 lb. |
| Columbo Root | 4 | 15 | 0.. | 6 | 0 | 0 C. |
| Cream of Tartar | 5 | 0 | 0.. | 5 | 15 | 0 — |
| Gallangal East-India | uncertain | | .. | 0 | 0 | 0 C. |
| Gentian Root | 3 | 10 | 0.. | 3 | 15 | 0 — |
| Ginseng | none | | .. | 0 | 0 | 0 lb. |
| Grains of Guinea | 6 | 10 | 0.. | 0 | 0 | 0 C. |
| Gum Ammo. Drop | none | | 0.. | 0 | 0 | 0 — |
| ——Lump | 5 | 0 | 0.. | 7 | 0 | 0 — |
| —— Animi | 3 | 10 | 0.. | 7 | 10 | 0 — |
| —— Arabic, E. I. | 2 | 0 | 0.. | 4 | 0 | 0 — |
| —— Turkey, Fine | 8 | 15 | 0.. | 10 | 10 | 0 — |
| —— Barbary | 5 | 5 | 0.. | 0 | 0 | 0 — |
| —— Assafœtida | 10 | 0 | 0.. | 15 | 0 | 0 — |
| —— Benjamin | 15 | 0 | 0.. | 60 | 0 | 0 — |
| —— Gambogium | 28 | 0 | 0.. | 30 | 0 | 0 — |
| —— Copal, scraped | 0 | 5 | 9.. | 0 | 6 | 0 lb. |
| —— Galbanum | 30 | 0 | 0.. | 0 | 0 | 0 C. |

| | £. | s. | d. | £. | s. | d per |
|---|---|---|---|---|---|---|
| Gum Guaiacum, from | 0 | 2 | 6 to | 0 | 3 | 0 lb. |
| —— Mastic | 0 | 4 | 3.. | 0 | 4 | 6 — |
| —— Myrrh | 15 | 0 | 0.. | 18 | 0 | 0 C. |
| —— Olibanum | 8 | 0 | 0.. | 11 | 0 | 0 — |
| —— Opponax | 80 | 0 | 0.. | 0 | 0 | 0 — |
| —— Sandrac | 8 | 0 | 0.. | 8 | 5 | 0 — |
| —— Senegal | 6 | 15 | 0.. | 7 | 0 | 0 — |
| —— Tragacanth | 27 | 0 | 0.. | 0 | 0 | 0 — |
| Jalap | 0 | 4 | 6.. | 0 | 4 | 9 lb. |
| Ipecacuanha | 0 | 14 | 6.. | 0 | 15 | 0 — |
| Isinglass Book | 0 | 9 | 0.. | 0 | 10 | 0 — |
| —— Leaf | 0 | 7 | 10.. | 0 | 8 | 6 — |
| —— Long Staple | 0 | 9 | 0.. | 0 | 10 | 0 — |
| —— Short Staple | 0 | 8 | 6.. | 0 | 9 | 6 — |
| Manna Flakey | 0 | 4 | 9.. | 0 | 5 | 0 — |
| —— Sicily in Sorts | 0 | 0 | 0.. | 0 | 0 | 0 — |
| Musk China | 0 | 14 | 0.. | 0 | 16 | 0 oz. |
| Nux Vomica | 2 | 0 | 0.. | 2 | 2 | 0 C. |
| Oil of Vitriol | 0 | 0 | 3¼ | 0 | 0 | 4 lb. |
| Opium, East-India | none | | .. | 0 | 0 | 0 — |
| —— Turkey | 1 | 4 | 0.. | 0 | 0 | 0 — |
| Pink Root | 0 | 4 | 0.. | 0 | 4 | 6 — |
| Quicksilver | 0 | 6 | 3.. | 0 | 6 | 6 — |
| Rhubarb, East-India | 0 | 9 | 0.. | 0 | 13 | 0 — |
| —— Russia | 0 | 18 | 0.. | 1 | 0 | 0 — |
| Saffron, Spanish | 2 | 10 | 0.. | 2 | 12 | 0 — |
| —— French | 1 | 18 | 0.. | 2 | 0 | 0 — |
| Sago | 6 | 15 | 0.. | 8 | 10 | 0 C. |
| Sal. Ammoniac | 10 | 10 | 0.. | 11 | 0 | 0 — |
| Sarsaparilla | 0 | 2 | 3.. | 0 | 4 | 9 lb |
| Sassafras | 7 | 10 | 0.. | 8 | 0 | 0 T. |
| Scammony, Aleppo | 1 | 9 | 0.. | 1 | 10 | 0 lb. |
| —— Smyrna | 0 | 18 | 0.. | 0 | 0 | 0 — |
| Senna | 0 | 4 | 6.. | 0 | 5 | 0 — |
| Seeds, Anni Alicant | 4 | 15 | 0.. | 5 | 0 | 0 C. |
| —— Coriander English | 0 | 14 | 0.. | 0 | 0 | 0 — |
| —— Cummin | 5 | 1 | 0.. | 0 | 0 | 0 — |
| —— Fenugreek | 1 | 15 | 0.. | 0 | 0 | 0 — |
| Shellack | 7 | 10 | 0.. | 10 | 15 | 0 — |
| Sticklack | 3 | 10 | 0.. | 7 | 0 | 0 — |
| Snake Root | 0 | 15 | 6.. | 0 | 0 | 0 lb. |
| Soap, Castile or Spanish | 9 | 0 | 0.. | 10 | 0 | 0 C. |
| Spermaceti, refined | 0 | 2 | 2.. | 0 | 0 | 0 lb. |
| Tamarinds, West-India | 9 | 0 | 0.. | 10 | 0 | 0 C. |
| Tapioca, Lisbon | 0 | 0 | 9.. | 0 | 1 | 0 lb. |
| Turmeric, Bengal | 4 | 10 | 0.. | 4 | 15 | 0 C. |
| —— China | 6 | 10 | 0.. | 7 | 0 | 0 — |
| —— West-India | 3 | 10 | 0.. | 4 | 4 | 0 — |
| Verdigris, Wet | 0 | 3 | 6.. | 0 | 0 | 0 lb. |
| —— Dry | 0 | 6 | 0.. | 0 | 6 | 6 — |
| —— Crystallized | 0 | 8 | 0.. | 0 | 8 | 6 — |
| Vitriol, Roman | 0 | 0 | 7.. | 0 | 0 | 0 — |
| —— Foreign white | 2 | 10 | 0.. | 0 | 0 | 0 C. |

*Price of Vials per Gross.*—8 oz. 70s.—6 oz. 58s.—4 oz. 47s.—3 oz. 43s.—2 oz. 36s.—1 oz. 30s. —½ oz. 24s.

Lisbon Leeches, 30s. per hundred.—True French ditto, 55s.

Essential Salt of Lemons, 4s. 6d. per dozen.

### METEOROLOGICAL REGISTER.

***From February the 23d, to March the 25th,*** **1815.**

**Kept by C. BLUNT, Philosophical Instrument Maker, No. 38, Tavistock-Street, Covent-Garden.**

| Moon. | Day. | Wind. | Barometrical Pressure. Max. | Min. | Mean. | Temperature. Max. | Min. | Mean. | |
|---|---|---|---|---|---|---|---|---|---|
| | 24 | NW | 30 | 30 | 30 | 54 | 41 | 47·6 | Fair |
| | 25 | NW | 30 | 29·98 | 29·99 | 53 | 42 | 48·3 | Fair |
| | 26 | NW | 30 | 30 | 30 | 54 | 41 | 48·3 | Fair |
| | 27 | NW | 30 43 | 30·33 | 30·395 | 50 | 30 | 43·3 | Fair |
| | 28 | NW | 30·39 | 30·28 | 30 31 | 46 | 36 | 41·3 | Fair |
| | 1 | SW | 30·19 | 30·11 | 30·145 | 54 | 39 | 45 | Fair |
| ◑ | 2 | SW | 30·18 | 30·18 | 30·18 | 50 | 37 | 45 6 | Rain |
| | 3 | SW | 30·16 | 30·12 | 30·14 | 50 | 39 | 43 | Fair |
| | 4 | S | 30·21 | 30·18 | 30·195 | 52 | 42 | 44 6 | Fair |
| | 5 | S | 30·19 | 30·19 | 30 19 | 55 | 43 | 48·3 | Cloudy |
| | 6 | SW | 30·18 | 30·13 | 30·167 | 55 | 40 | 47 | Fair |
| | 7 | SW | 29·9 | 29·75 | 29·807 | 51 | 41 | 44 | Cloudy |
| | 8 | W | 29·46 | 29·37 | 29·397 | 50 | 38 | 46 | Stormy & Rain |
| | 9 | W | 29 37 | 29 30 | 29·325 | 48 | 34 | 43 | Rain |
| | 10 | NW | 29 56 | 29·42 | 29·477 | 49 | 32 | 39 6 | Rain |
| ● | 11 | NW | 29·56 | 29·41 | 29 49 | 48 | 33 | 42 6 | Fair |
| | 12 | NW | 28·95 | 28 95 | 28 95 | 49 | 37 | 44 | Fair |
| | 13 | NW | 29 19 | 28 92 | 29·035 | 50 | 34 | 44 | Fair wind, hail, & thunder at 5 p m. |
| | 14 | NW | 29·85 | 29·59 | 29·682 | 50 | 33 | 42·3 | Rain |
| | 15 | W | 29 93 | 29 83 | 29 892 | 55 | 40 | 46 6 | Rain |
| | 16 | SW | 29 85 | 29 68 | 29 773 | 61 | 44 | 53 | Rain |
| | 17 | NW | 29·97 | 29·91 | 29·937 | 56 | 44 | 49 | Wind Fair |
| ◐ | 18 | NW | 30 03 | 30 | 30 012 | 55 | 42 | 48 3 | Rain |
| | 19 | NW | 30· | 30 | 30 | 56 | 48 | 50 6 | Cloudy |
| | 20 | SW | 29·91 | 29 87 | 29·817 | 60 | 44 | 52 6 | Cloudy |
| | 21 | SW | 29·79 | 29·55 | 29·685 | 60 | 48 | 54·3 | Cloudy. Rain. |
| | 22 | W | 29·6 | 29 55 | 29·576 | 59 | 45 | 52·3 | Stormy Wind |
| | 23 | W | 29 37 | 29 25 | 29·337 | 58 | 44 | 53 | Wind & Rain |
| | 24 | W | 29·30 | 29 30 | 29·30 | 57 | 44 | 50·3 | Wind & Rain |
| ○ | 25 | W | 29 41 | 29 32 | 29 37 | 57 | 47 | 49 6 | Wind & Rain |

RESULTS.

| | | | |
|---|---|---|---|
| Mean barometrical pressure of the month | 29·7867 | Mean temperature of the month | 46·575 deg. |
| Maximum 30·43 | wind at NW | Maximum 61, | wind at SW |
| Minimum 28·92 | ——— NW | Minimum 30, | ——— NW |

*Scale exhibiting the prevailing Winds during the Month.*

| N | NE | E | SE | S | SW | W | NW |
|---|---|---|---|---|---|---|---|
| 0 | 0 | 0 | 0 | 2 | 8 | 7 | 13 |

| | Mean barometrical pressure. | Mean temperature. |
|---|---|---|
| From the full moon on the 23d Feb. to the last quarter on the 2d March | 30·145 | 45·63 |
| ——— last quarter on the 2d, to the new moon on the 11th | 29·793 | 44·23 |
| ——— new moon on the 11th, to the first quarter on the 18th | 29·611 | 46·74 |
| ——— first quarter on the 18th, to the new moon on the 25th | 29·593 | 51·8 |

